# Melatonin Suppresses Hypoxia-Induced Migration of HUVECs via Inhibition of ERK/Rac1 Activation

**DOI:** 10.3390/ijms150814102

**Published:** 2014-08-13

**Authors:** Ling Yang, Jianchao Zheng, Rui Xu, Yujie Zhang, Luo Gu, Jing Dong, Yichao Zhu, Ruijue Zhou, Lu Zheng, Xiaoying Zhang, Jun Du

**Affiliations:** 1 Department of Cardiology, the Third Affiliated Hospital of Soochow University, Changzhou 213003, Jiangsu, China; E-Mails: Linda_YL73@yahoo.com (L.Y.); ruijuezhoucz@yahoo.com (R.Z.); 2 Department of Physiology, Nanjing Medical University, Nanjing 210029, Jiangsu, China; E-Mails: zhjch@njmu.edu.cn (J.Z.); xurui20062624@gmail.com (R.X.); zhangyujie@njmu.edu.cn (Y.Zhang); lgu@njmu.edu.cn (L.G.); zhuyichao@njmu.edu.cn (Y.Zhu); 3 Epidemiology and Biostatistics and Ministry of Education (MOE) Key Lab for Modern Toxicology, Nanjing Medical University, Nanjing 210029, Jiangsu, China; E-Mail: cindydongjing@gmail.com; 4 Comprehensive Laboratory, the Third Affiliated Hospital of Soochow University, Changzhou 213003, Jiangsu, China; E-Mail: luzhengcz@yahoo.com

**Keywords:** hypoxia, ERK, Rac1, HIF-1α, migration, HUVEC, melatonin

## Abstract

Melatonin, a naturally-occurring hormone, possesses antioxidant properties and ameliorates vascular endothelial dysfunction. In this study, we evaluate the impact of melatonin on the migratory capability of human umbilical vein endothelial cells (HUVECs) to hypoxia and further investigate whether ERK/Rac1 signaling is involved in this process. Here, we found that melatonin inhibited hypoxia-stimulated hypoxia-inducible factor-1α (HIF-1α) expression and cell migration in a dose-dependent manner. Mechanistically, melatonin inhibited Rac1 activation and suppressed the co-localized Rac1 and F-actin on the membrane of HUVECs under hypoxic condition. In addition, the blockade of Rac1 activation with ectopic expression of an inactive mutant form of Rac1-T17N suppressed HIF-1α expression and cell migration in response to hypoxia, as well, but constitutive activation of Rac1 mutant Rac1-V12 restored HIF-1α expression, preventing the inhibition of melatonin on cell migration. Furthermore, the anti-Rac1 effect of melatonin in HUVECs appeared to be associated with its inhibition of ERK phosphorylation, but not that of the PI3k/Akt signaling pathway. Taken together, our work indicates that melatonin exerts an anti-migratory effect on hypoxic HUVECs by blocking ERK/Rac1 activation and subsequent HIF-1α upregulation.

## 1. Introduction

Melatonin, chemically known as *N*-acetyl-5-methoxytryptamine, is a naturally-occurring hormone that is secreted mainly in the pineal gland, gastrointestinal tract, skin and retina. Besides its function as a synchronizer of the biological clock, melatonin possesses antioxidant properties and helps ameliorate vascular endothelial dysfunction [1,2]. Hypoxia is a characteristic feature of many physiological or pathological processes driving endothelial cells (ECs) to migrate and form new vasculature, and enhanced endothelial cell motility plays a pivotal role in promoting angiogenesis [3]; however, the role of melatonin in regulating ECs’ motility under hypoxic condition is largely unknown.

Induction of hypoxia-inducible factor-1α (HIF-1α) is well known as a hallmark feature of hypoxia. Under hypoxia, HIF-1α translocates into the nucleus, where it upregulates the transcription of its target genes, such as *MMPs*, *VEGF*, as well as *VE-cadherin*, which are closely correlated with ECs’ migration and angiogenesis [4,5,6]. A lot of evidence indicates that melatonin is a potent HIF-1α stabilization inhibitor in various types of cells [7,8,9], although some reports described that this hormone may induce the transcriptional activity of HIF-1α in hypoxia [10,11]. Thus, the mechanism underlying the effect of melatonin on HIF-1α expression in ECs remains to be determined.

Rac1, a member of the Rho GTPases family, has emerged as a critical regulator of cell migration, since it has a role in cytoskeleton organization. It is revealed that inhibition of Rac1 protects against hypoxia/reoxygenation-induced lipid peroxidation in human umbilical vein endothelial cells (HUVECs) [12]. There is also evidence that endothelial Rac1 is essential for endothelium-dependent vasomotor response and ischemia-induced angiogenesis by using endothelial-specific Rac1 haploinsufficient (EC-Rac1(+/−)) mice [13]. A previous study has demonstrated that melatonin plays a crucial role in decreasing the permeability of monolayer endothelial cells induced by IL1β [14]. However, it is not known whether the effect of melatonin on HUVECs is dependent on Rac1 or not. Recently, the modulatory effect of melatonin on RhoA, another member of the Rho family, has been described. Ortiz-Lopez *et al.* reported that inactivation of the RhoA/ROCK pathway is implicated in the inhibitory effects of melatonin on cell migration by changing cytoskeletal organization in MCF-7 cells [15]. It should be noted that RhoA/ROCK may have an antagonist action on Rac1 [16,17]. Hence, it is worthwhile to determine the role of melatonin on the regulation of Rac1 that could be important in regulating ECs’ migration. In addition, concomitant activation of Rac1, ERK and PI3K is observed after hepatocyte growth factor (HGF) stimulation, which is required to achieve the capacity of neuron migration [18]. PI3K and ERK have also been identified as potent modulators of HUVECs growth inhibition by melatonin [19]. Therefore, we presume that melatonin regulates Rac1 activation and cell migration by mediating ERK and/or PI3K signaling.

In the present study, we investigated the relationship between PI3K, ERK, Rac1 and cell migration response to hypoxia in HUVECs and provide evidence that melatonin blocked Rac1 activation and disrupted the stabilization of its downstream effector HIF-1α, leading to a marked inhibition of the migration of HUVECs in response to hypoxia. Furthermore, our results suggested that the anti-Rac1 effect of melatonin in HUVECs is associated with its inhibition of ERK, but not PI3K activation.

## 2. Results and Discussion

### 2.1. Melatonin Inhibits Hypoxia-Induced HUVEC Migration in Vitro

**Figure 1:**
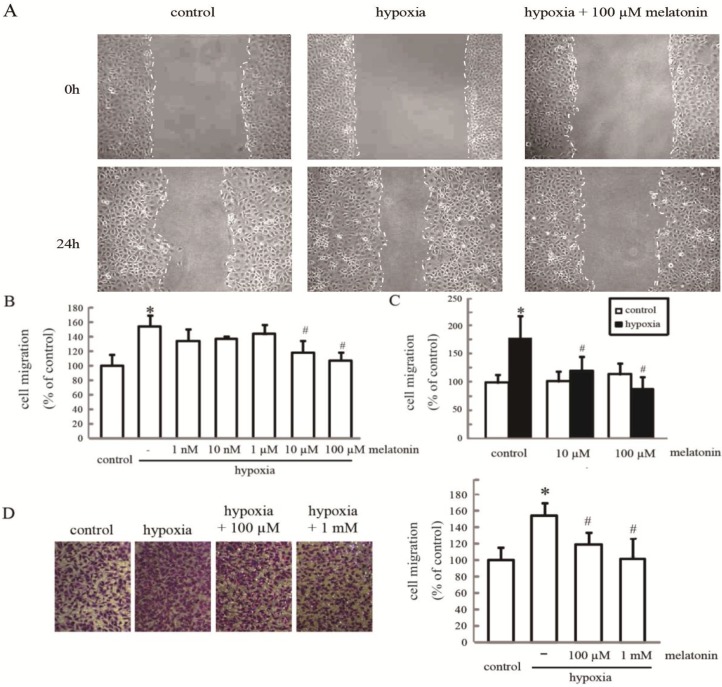
The effect of melatonin on hypoxia-induced HUVEC migration. (**A**) HUVECs were incubated with or without melatonin at the indicated concentrations and then exposed to hypoxia for 24 h. A representative wound healing assay is presented. The gap is indicated by white dotted lines; (**B**,**C**) The relative cell migration rate was determined using the wound closure assay. The values are presented as the mean ± SD of three independent experiments; and (**D**) Cell migration was assessed by the Transwell migration assay in HUVECs incubated with 0.1 and 1 mM melatonin and then exposed to hypoxia for 3 h. * *p* < 0.05, referring to the difference between cells treated with and without hypoxia; ^#^ *p* < 0.05, referring to the difference between cells treated with melatonin and hypoxia relative to the cells exposed to hypoxia alone.

Hypoxia is a characteristic feature of many human physiological and pathological processes. To assess the effect of melatonin on hypoxia-induced HUVEC migration, we treated cells with different doses of melatonin [8,20] and measured the migration rate by wound closure assay. As shown in Figure 1A, an upregulation of HUVEC migration occurred after exposure to hypoxia; the cell migratory ability decreased gradually with increasing doses of melatonin. Statistical analysis showed that melatonin at 10–100 µM significantly suppressed hypoxia-stimulated cell migration (*p* < 0.05) (Figure 1B,C), while the migratory rate was not altered significantly in melatonin (10–100 µM)-treated cells compared to the control cells (Figure 1C). To preclude the possibility that the melatonin reduced cell migration was associated with decreased cell proliferation, we also assessed the migration rate of HUVECs using Transwell assays under either normoxic or hypoxic conditions for 3 h. Hypoxic cells (2 × 10^5^/well) exhibited a significantly increased capacity to penetrate the chamber filter, which was reversed by melatonin (100 µM–1 mM) pretreatment. These results showed that melatonin suppressed hypoxia-increased migration of HUVECs *in vitro*.

### 2.2. Melatonin Inhibits Hypoxia-Induced Rac1 Activation

**Figure 2:**
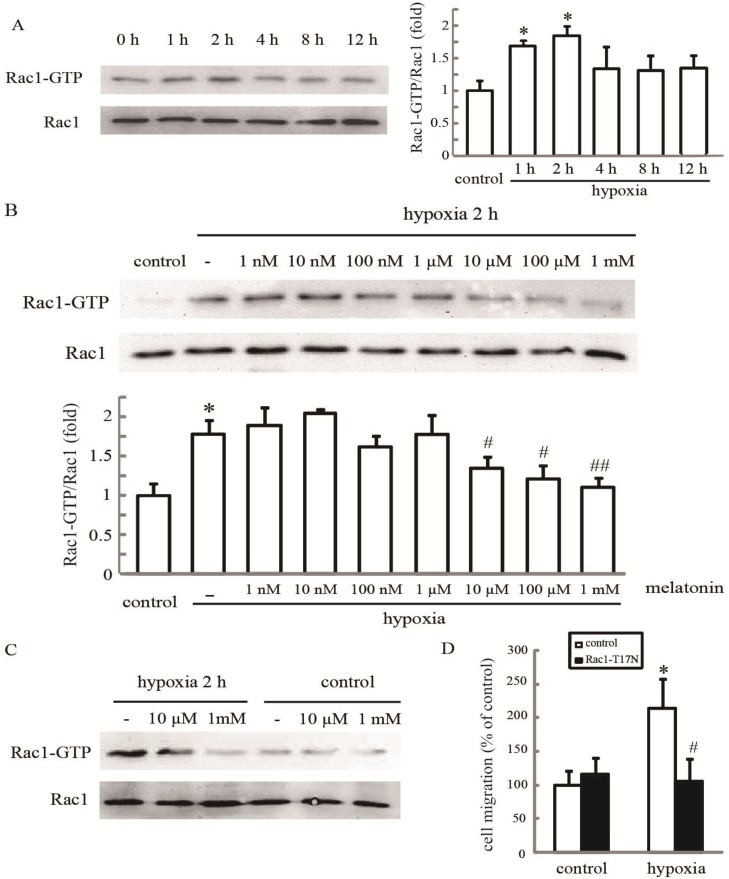
Melatonin inhibits hypoxia-induced Rac1 activation. (**A**) The effect of hypoxia on Rac1 activation. HUVECs were incubated under normoxia or hypoxia for the indicated periods, and cellular lysates were assayed for active Rac1 by pull-down assay; (**B**,**C**) The effect of melatonin on hypoxia-induced Rac1 activation. After treatment with melatonin at the indicated concentrations, cells were exposed to hypoxia for 2 h and then analyzed for Rac1 activation. * *p* < 0.05, referring to the difference between cells treated with and without hypoxia; ^#^ *p* < 0.05, ^##^ *p* < 0.01, referring to the difference between cells treated with melatonin and hypoxia relative to the cultures exposed to hypoxia alone; and (**D**) The inactive mutant of Rac1-T17N inhibited hypoxia-mediated migration. Cells transfected with an empty vector or Rac1-T17N were grown under hypoxia for 24 h, and the cell migration rate was determined by the wound healing assay. * *p* < 0.05, referring to the difference between cells treated with and without hypoxia; ^#^ *p* < 0.05, referring to the difference between hypoxic cells transfected with Rac1-T17N and empty vector.

Rac1 can be activated by hypoxia in some kinds of tumor cells [21,22]. Therefore, we examined whether endogenous Rac1 activation also occurred in our system. The results showed that hypoxia induced an increase in Rac1-GTP within 1–2 h, while the level of total Rac1 protein in HUVECs remained unmodified at all of these time points (Figure 2A). Then, HUVECs were treated with different doses of melatonin (1 nM–1 mM) for 24 h, and then exposed to hypoxia for 2 h. Western blot results indicated that the increased Rac1 activation by temporary hypoxia exposure was inhibited by melatonin dose-dependently. As expected, melatonin at a 10 µM–1 mM concentration significantly suppressed hypoxia-stimulated Rac1 activation (*p* < 0.05) (Figure 2B,C).

To further determine whether Rac1 is involved in melatonin-mediated cell migration, HUVECs were transfected with either an empty vector or a Rac1-T17N (inactive mutant of Rac1) expression vector, then stimulated with hypoxia stress for 24 h, and the cell migration was examined. We found that, in cells transfected with the empty vector, the cell migration rate was increased significantly after the hypoxia. However, in cells transfected with the Rac1-T17N expression vector, such a stimulatory effect of hypoxia on cell migration was eliminated (Figure 2D). Together, these findings indicate that melatonin-mediated inhibition of hypoxic cell migration acts through blocking of Rac1 activation.

### 2.3. Melatonin Inhibits Hypoxia-Induced Rac1 Redistribution

**Figure 3:**
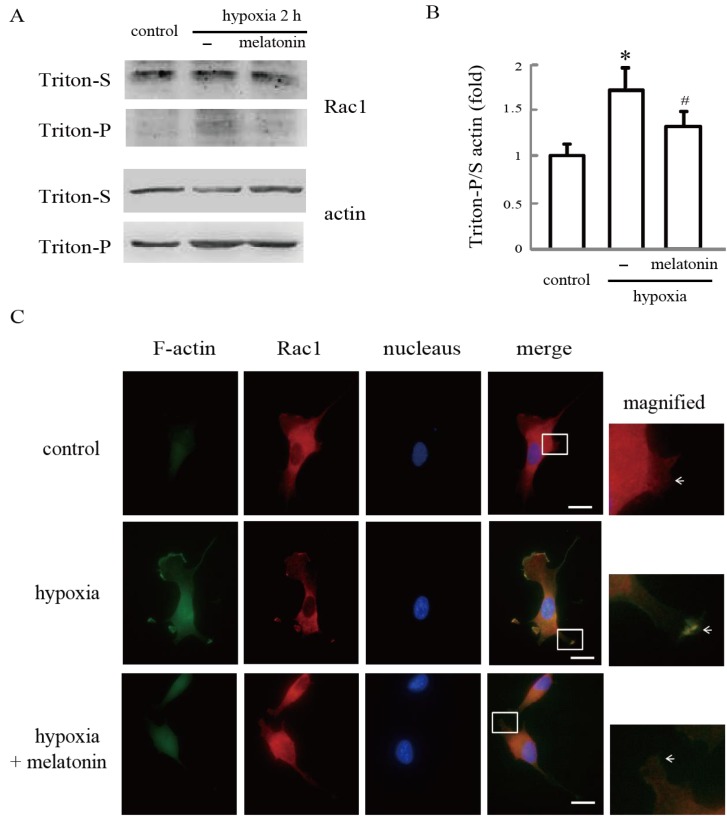
Melatonin inhibits hypoxia-induced Rac1 redistribution. (**A**,**B**) Redistribution of Rac1 and actin during hypoxia. After being treated with or without melatonin for 24 h, HUVECs were incubated under normoxia or hypoxia for 2 h. Rac1 and actin in equal amounts of Triton X-100-soluble and -insoluble fractionswere detected by western blotting. Each bar graph represents the mean ± SD of three independent experiments. * *p* < 0.05, referring to the difference between cells treated with and without hypoxia; ^#^ *p* < 0.05, referring to the difference between cells treated with melatonin and hypoxia relative to the cultures exposed to hypoxia alone; and (**C**) Melatonin reverses hypoxia-stimulated Rac1 translocation in HUVECs. After pretreatment with 100 µM of melatonin for 24 h, cells were grown under hypoxia for 2 h and stained for Rac1 (red) and F-actin (green), as described in the Materials and Methods Section. Cells were counterstained with DAPI (blue). Images are representative of at least three independent determinations. The illustrations in the right side column show the merged magnified images in the white border boxes, white arrows point to the co-localization of Rac1 and F-actin on the membrane of HUVECs. Magnification, 400×. Scale bars, 10 µm.

Since Rac1-regulated cell migration is not solely dependent on its activation status, but its redistribution to the extensions of the plasma membrane also contributes to its activity [17,23,24], we also examined Rac1 localization in cultured hypoxic cell after melatonin treatment. Western blot analysis revealed that the hypoxia stress significantly increased the ratio of insoluble/soluble actin. More importantly, the increases of the ratio were much less in the same treated cells incubated with melatonin (Figure 3A,B). In addition, hypoxia treatment partially enhanced translocation of Rac1 from the cytosolic fraction (soluble part) to the cytoskeletal fraction (insoluble part); however, this translocation was reversed by melatonin in HUVECs.

We also performed immunofluorescence staining to investigate the distribution pattern of Rac1 and filamentous actin (F-actin). Our results revealed that phalloidin staining of F-actin in untreated cells was weak, and large amounts of Rac1 were localized in the cytoplasm. However, after exposure to hypoxia for 2 h, F-actin abundance was obviously increased, and substantial Rac1 and F-actin co-localization was observed especially in the periphery of the cells. Treatment with melatonin before hypoxia not only disrupted the formation of F-actin fibers, but also prevented an increase in the fluorescence intensity of co-localized Rac1 and F-actin on the membrane of HUVECs (Figure 3C).

### 2.4. Rac1 Is Required for Melatonin-Reduced HIF-1α Expression and Cell Migration in Hypoxia

**Figure 4:**
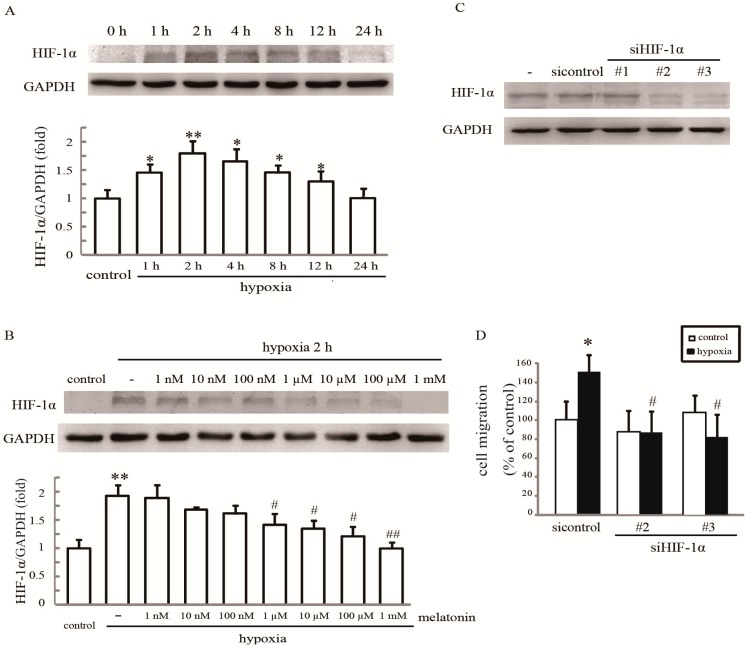
Melatonin inhibits hypoxia-induced cell migration by suppressing HIF-1α. (**A**) The effect of hypoxia on HIF-1α expression. Cells were incubated under normoxia or hypoxia for the indicated periods. Cell lysates were assayed for HIF-1α expression; (**B**) The effect of melatonin on hypoxia-induced HIF-1α expression. After treatment with the indicated concentrations of melatonin, cells were exposed to hypoxia for 2 h and then analyzed for Rac1 activation. * *p* < 0.05, ** *p* < 0.01, referring to the difference between cells treated with and without hypoxia; ^#^ *p* < 0.05, ^##^ *p* < 0.01, referring to the difference between cells treated with melatonin and hypoxia relative to the cultures exposed to hypoxia alone; (**C**) The effect of siRNA on the intracellular levels of HIF-1α. Total protein extracts from cells transfected with HIF-1αsiRNA or control siRNA (mock) were analyzed by western blotting for HIF-1α. GAPDH was used a loading control; and (**D**) HIF-1α silencing inhibits cell migration. Cells transfected with 60 nmol·L^−1^ HIF-1α siRNA were grown under hypoxia for 24 h, and the cell migratory rate was examined by wound healing assays. * *p* < 0.05, referring to the difference between cells treated with and without hypoxia; ^#^ *p* < 0.05, referring to the difference between hypoxic cells transfected with HIF-1α siRNA and control siRNA.

The stabilization of endogenous HIF-1α is a hallmark of hypoxia, so we first examined whether HIF-1α activation also occurred in our system. Western blotting analysis showed that the amount of HIF-1α was increased significantly from 1 h after hypoxia stimulation with maximal activation at 2 h. However, after 24 h, it declined toward basal levels (Figure 4A). As shown in Figure 4B, HIF-1α expression was reversed by melatonin pretreatment in hypoxia-treated cells dose-dependently (Figure 4B), indicating that melatonin has a significant influence on HIF-1α expression in response to hypoxia. Two HIF-1α siRNAs independently knocked down HIF-1α expression by more than 80%, as assessed by western blot analysis (Figure 4C), and resulted in a significant reduction of hypoxia-induced cell migration (Figure 4D).

**Figure 5:**
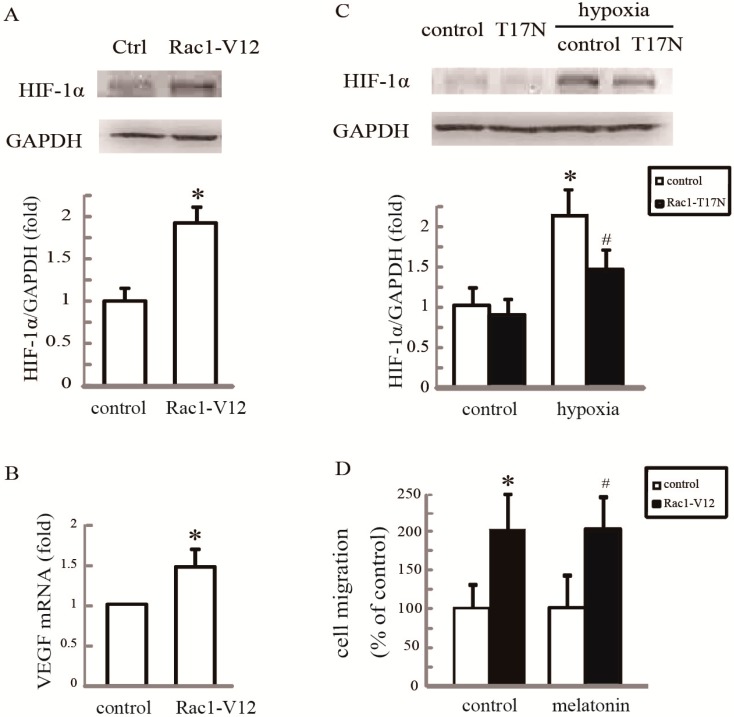
Rac1 is required for melatonin-reduced HIF-1α expression in hypoxic HUVECs. (**A**)Overexpression of Rac1-V12 promoted HIF-1αexpression. Cells were transfected with empty vector or Rac1-V12, and western blotting was performed to examine HIF-1α expression; (**B**) A q-PCR assay was performed to examine VEGF mRNA. * *p* < 0.05, referring to the difference between cells transfected with Rac1-V12 and empty vector; (**C**) Overexpression of Rac1-T17N inhibited hypoxia-mediated HIF-1α expression. Cells transfected with an empty vector or Rac1-T17N were grown under hypoxia for 2 h, and HIF-1α expression was examined by western blotting. * *p* < 0.05, referring to the difference between cells treated with and without hypoxia; ^#^ *p* < 0.05, referring to the difference between hypoxic cells transfected with Rac1-T17N and empty vector; and (**D**) Melatonin failed to affect migration of cells overexpressing Rac1-V12. Cells transfected with an empty vector or Rac1-V12 were treated with 100 µM melatonin, and the cell migration rate was determined by wound closure assay. * *p* < 0.05, referring to the difference between cells transfected with Rac1-V12 and empty vector; ^#^ *p* < 0.05, referring to the difference between cells transfected with Rac1-V12 plus melatonin relative to the cultures with empty vector plus melatonin.

We next examined whether, specifically, inactivation of Rac1 was responsible for the inhibition of cell motility by the suppression of HIF-1α. Cells were transfected with either empty vectors or vectors encoding the active mutant of Rac1-V12. As shown in Figure 5A,B, the active mutant of Rac1-V12 markedly increased the protein levels of HIF-1α and its target gene, *VEGF*. Additionally, we examined the effect of Rac1-T17N on hypoxia-induced HIF-1α expression. As shown in Figure 5C, Rac1-T17N significantly attenuated the increased level of HIF-1α protein under hypoxia. As Rac1 activation is required for HIF-1α expression, we asked if Rac1 activation was sufficient to counteract the inhibitory effect of melatonin on cell migration. Indeed, overexpression of Rac1-V12 in HUVECs antagonized the blockade of migration by melatonin (Figure 5D). Collectively, these results support the conclusion that Rac1 inactivation is required for melatonin-reduced HIF-1α expression and cell migration.

### 2.5. Melatonin Reverses Hypoxia-Activated Rac1 Activation Mediated through ERK

**Figure 6:**
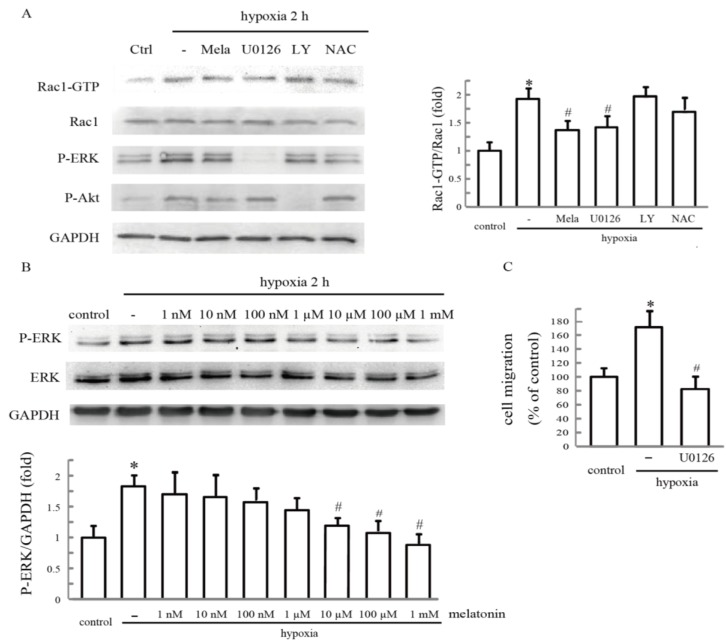
Melatonin reverses hypoxia-activated Rac1 activation and cell migration mediated through ERK. (**A**) The effect of PI3K, ERK and ROS on hypoxia-stimulated Rac1 activation. After treatment with 10 µM LY294002, or 10 µM U0126, or 1 mM NAC for 1 h, cells were incubatedunder hypoxia for 2 h, and cell lysates were analyzed forRac1 activation by pull-down assays. Each bar graph represents the mean ± SD of three independent experiments. * *p* < 0.05, referring to the difference between cells treated with and without hypoxia; ^#^ *p* < 0.05, referring to the difference between cells treated with hypoxia plus melatonin or U0126 and relative to the cells exposed to hypoxia alone; (**B**) The effect of hypoxia on ERK activation. Cells were incubated under normoxia or hypoxia for the indicated time. Cellular lysates were assayed for ERK phosphorylation; and (**C**) The effect of ERK inhibitor U0126 on hypoxia-stimulated cell migration. After pretreatment with 10 µM U0126 for 1 h, cells were exposed to hypoxia for 24 h, and the cell migration rate was determined by wound closure assay. * *p* < 0.05, referring to the difference between cells treated with and without hypoxia; ^#^ *p* < 0.05, referring to the difference between cells treated with U0126 plus hypoxia relative to the cells exposed to hypoxia alone.

Previous reports have shown that ROS, ERK and PI3K play vital roles in Rac1 activation. To determine whether these factors are involved in hypoxia-mediated Rac1 activation in HUVECs, cells were pretreated with melatonin for 24 h, or ERK inhibitor U0126, PI3K inhibitor LY294002 or ROS scavenger *N*-acetyl-cysteine (NAC) separately for 30 min, and the expression levels of Rac1 under hypoxia were measured. Unlike NAC and LY294002, U0126 significantly decreased Rac1 activation in hypoxic HUVECs (Figure 6A). We then treated the cells with melatonin and examined ERK activities after stimulation with hypoxia. Melatonin treatment resulted in the inhibition of ERK activity under hypoxia in a dose-dependent manner, as determined by western blotting with an antibody against the phosphorylated form of ERK (Figure 6B). To further determine whether hypoxia stimulated endothelial cell migration in an ERK-dependent manner, we investigated the effect of U0126 on cell migration using a wound closure assay. Pretreatment with U0126 resulted in a remarkable inhibition of hypoxia-promoted cell migration (Figure 6C). These results indicate that ERK activation lies upstream of Rac1, and melatonin inhibits hypoxia-induced Rac1 activation and cell migration by blocking ERK activation.

### 2.6. Discussion

Epidemiological studies suggest an association between decreased melatonin level and the increased chance of cardiovascular diseases [25,26]. Melatonin has been shown to ameliorate hypertension in animal models and patients [27,28]. In addition, melatonin may decrease the secretion of VEGF in cancer cells and exert antiangiogenic actions [29,30,31]. Interestingly, long-term melatonin treatment has proven the lack of any noteworthy side effects [32,33]. Thus, melatonin may be of potential use in the prevention and treatment of cardiovascular diseases and some types of cancer. In our system, the HUVEC migration rate was accelerated under hypoxia; however, it was suppressed by melatonin dose-dependently. Therefore, melatonin directly inhibits the migratory capability of hypoxic HUVECs, which is a critical step for angiogenesis. Based on this, the mechanism underlying the effect of melatonin on decreasing HUVECs migration under hypoxia was investigated.

In the present study, we found that hypoxia triggers a rapid activation of Rac1. Several studies have reported that Rac1 activation promotes angiogenesis [34,35], and its inactivation has been shown to be involved in the mechanism of Akt against ischemic brain injury [36]. Rac1 is also emerged as a critical regulator of cytoskeletal rearrangement and mediates EGF-stimulated cancer cell migration [37]. Rac1 activation has been shown to be important in promoting the cutaneous wound healing process [38] and is linked to the progression of murine yolk sac vascular remodeling *in vivo* [39]. Consistent with these reports, our results showed that when Rac1 activity was blocked by Rac1-T17N, hypoxia-stimulated cell migration was dramatically diminished, while Rac1-V12 increased the cell migration rate in HUVECs, suggesting that Rac1 activation serves as a mediator of hypoxia-stimulated HUVEC migration. Furthermore, we observed that melatonin caused a marked downregulation of Rac1 activation in a dose-dependent manner, and restoring Rac1 activation in HUVECs significantly antagonized the blockade of migration by melatonin. Therefore, our results suggest that melatonin reduces hypoxia-induced cell migration, at least in part through the inhibition of Rac1 activation.

Rac1 plays a central role in the key intracellular signal transduction pathway for the regulation of the polymerization of preexisting filaments in the leading edge, as well as inducing the rapid formation of filopodia, which mediate the migratory and invasive abilities of different types of cells [17,23,24]. Here, we observed that hypoxia not only induced Rac1 redistribution from the cytoplasm to the cytoskeleton, but also increased actin polymerization. Inhibiting Rac1 activation by melatonin rescued Rac1 location and actin cytoskeleton remodeling under hypoxia. Melatonin also abrogated the hypoxia induced co-location of Rac1 and filamentary actin (F-actin) on filopodia. Interestingly, we noticed that a large amount of Rac1 still exists in the cytoplasm, regardless of hypoxia or melatonin stimulation; hence, Rac1 may possess other properties in regulating migration besides its function in cytoskeleton reorganization in filopodia, and melatonin may reduce migration via inhibiting another downstream target of Rac1.

The association between Rac1 activation and HIF-1α upregulation during hypoxia has been reported recently [40]. In fact, HIF-1α has been identified as a novel prognostic biomarker, initiating the transcription of its target genes, which are closely correlated with ECs’ migration under hypoxic condition [6,8], and the loss of HIF-1α in ECs results in the inhibition of angiogenesis in solid tumors [41]. Similar to these findings, our results found that when HIF-1α signaling was blocked, hypoxia-stimulated cell migration was dramatically diminished. Adding to the results that melatonin reduced HIF-1α expression and this reduction is associated with its reversal of increased cell migration under hypoxia, these findings suggest that melatonin, through its regulation of HIF-1α, serves as a suppressor of hypoxia-stimulated HUVEC migration. Moreover, the overexpression of Rac1-V12 has a similar stimulatory effect on HIF-1α expression, while Rac1 inactivation decreases HIF-1α in HUVECs. Consequently, we conclude that Rac1 is an upstream regulator of the HIF-1α, and melatonin may suppress hypoxia-stimulated HUVEC migration via the Rac1/HIF-1α pathway.

We next examined the involved mechanism of melatonin regulating Rac1 in our system. The PI3K and ERK signaling pathways have been shown to control HIF-1α expression [42,43]. A previous study reported that notoginsenoside Ft1 promotes VEGF release and angiogenesis in HUVECs via the PI3K/Akt and Raf/MEK1/ERK pathways [44]. Our results revealed that hypoxia elevated not only the level of PI3K/Akt activity, but also ERK phosphorylation in HUVECs. However, specific inhibitors for ERK, but not PI3K, reduce hypoxia-induced Rac1 activity. Our results are similar with the finding by Liu *et al.*, who demonstrated that melatonin induced cellular cycle arrest by preventing ERK activation, but not Akt, P38 or JNK activation in human osteoblastic cells [45]. Our results showed that melatonin dose-dependently decreased ERK activity in hypoxic HUVECs, and the ERK inhibitor not only suppressed Rac1 activation, but also reduced cell migration by hypoxia; therefore, it may be reasonable to speculate that ERK inhibition is involved in melatonin-induced Rac1 inactivation.

The mechanism whereby Rac1 regulates HIF-1α expression in this study remains to be elucidated. Increasing studies have demonstrated that melatonin has antioxidant properties [2,46,47]. Furthermore, melatonin exhibits an inhibitory influence on ROS production induced by hypoxia, leading to the destabilization of HIF-1α protein and a decreased expression of VEGF in colon cancer cells [7]. Notably, Rac1 is capable of binding and activating NADPH oxidase [48,49,50], which is involved in the control of intracellular redox status and angiogenesis by inducing ROS production [34,51]. Therefore, it might be reasonable that melatonin led to a marked inhibition of HIF-1α expression through downregulation of Rac1 activity under hypoxia. Although a limitation of our current study is the lack of *in vivo* data, these above studies, together with our findings, suggest that the ERK/Rac1/HIF-1α signal transduction pathway may play a key role in the regulation of HUVEC migration by melatonin.

## 3. Experimental Section

### 3.1. Cell Culture and Transfection

HUVECs were purchased from the American Type Culture Collection (ATCC) (Rockville, MD, USA) and maintained in high glucose Dulbecco’s modified Eagle’s medium (DMEM) (Gibco, Grand Island, NY, USA) supplemented with 10% (*v*/*v*) fetal bovine serum (FBS, Hyclone, Logan, UT, USA), 100 U/mL penicillin, 100 µg/mL streptomycin and cultured at 37 °C in a humidified atmosphere. For growth under hypoxia, cells were incubated at 37 °C in a modular chamber flushed with 1% O_2_, 5% CO_2_ and 94% N_2_. To reduce the risk of the HUVECs’ functional properties in an *in vitro* environment, they were used for no more than five passages. Before treatment, cells were made quiescent by serum starvation overnight, as previously described [52]. For drug studies, 10 µM LY294002 (Sigma, St. Louis, MO, USA), or 10 µM U0126 (Alexis, Lausen, Switzerland), or 1 mM NAC (Sigma) were added to the medium for 1 h before hypoxia.

Full-length Rac1-T17N and Rac1-V12 (kindly provided by Dr. Shoshana Ravid, the Hebrew University, Jerusalem, Israel) were cloned into the pEGFP-N1 vector. Cells, when approximately 80% confluent and were transfected with empty vector or pEGFP-N1 expressing Rac1-T17N or Rac1-V12 using Lipofectamine 2000, as instructed by the manufacturer (Invitrogen, Carlsbad, CA, USA). The sequences of small interfering RNA (siRNA) for HIF-1α were as follows: #1, 5'-CCACCACUGAUGAAUUAAATT-3', #2, 5'-GCUGGAGACACAAUCAUAUTT-3', and #3, 5'-GCCGCUCAAUUUAUGAAUATT-3'; and the sequence of scrambled siRNA was 5'-UUCUCCGAACGUGUCACGUTT-3' (GenePharma Co., Shanghai, China). Cells were transfected with scrambled siRNA or Rac1 siRNA with Lipofectamine 2000, according to the manufacturer’s instruction.

### 3.2. In Vitro Wound Closure Assay

For the cell migration assay, the cell wound closure assay was performed as previously described [20]. Briefly, a scratch was made in the monolayer of HUVEC cells by using a 10-µL pipette tip. The cell culture was washed with PBS to remove non-adherent cells and incubated in medium containing the indicated concentrations of melatonin or other inhibitors, then exposed to hypoxia or normoxia for 24 h. Wound healing was photographed microscopically.

### 3.3. Transwell Migration Assay

Logarithmically growing cells were harvested, washed and suspended in DMEM without FBS. Cells (2 × 10^5^) were seeded into polycarbonate membrane inserts (8-µm pore size) in 24-Transwell cell culture dishes (Corning, NY, USA). Cells were allowed to attach to the membrane for 30 min before hypoxia. The lower chamber was filled with 800 µL DMEM with 10% FBS as a chemoattractant. Cells were permitted to migrate for 3 h in hypoxia. After incubation, stationary cells were removed from the upper surface of the membranes. The cells adhering to the lower surface were stained with 0.1% crystal violet. The number of stained cells was counted with the help of Image J software.

### 3.4. Total Cell Extraction

Subconfluent cells were washed twice with PBS and then lysed with ice-cold RIPA lysis buffer (50 mM Tris, 150 mM NaCl, 1% Triton X-100, 1% sodium deoxycholate, 0.1% SDS, 1 mM sodium orthovanadate, 1 mM sodium fluoride, 1 mM EDTA, 1 mM PMSF and 1% cocktail of protease inhibitors), pH 7.4. The lysates were then centrifuged and the supernatants collected for immunoblotting assay.

### 3.5. Extraction of the Triton X-100-Soluble and -Insoluble Fractions

The procedure was performed as previously described [53]. The treated subconfluent cells were washed twice with PBS, then lysed with ice-cold Triton lysis buffer (0.5% Triton X-100, 300 mM sucrose, 5 mM Tris-Cl, 2 mM EDTA, 1% cocktail of protease inhibitors, 1 mM Na_3_VO_4_ and 0.5 mM PMSF), pH 7.4. After 20 min of centrifugation at 12,000× *g*, the supernatant was saved as the Triton X-100 soluble fractions. The pellet was then suspended in sample buffer, referred to as Triton X-100 insoluble fractions. Then, the shift between detergent-soluble and detergent-insoluble fractions of Rac1 and actin were assessed by SDS-PAGE and western blot analysis.

### 3.6. SDS-PAGE and Western Blot Analysis

Total protein content was determined using the BCA assay, and immunoblotting assays were performed as described previously [53]. The following antibodies were used: rabbit anti-HIF-1α antibody, rabbit anti-phospho-Akt (Ser473) antibody (Cell Signaling, Beverly, MA, USA), mouse anti-ERK antibody and goat anti-phospho-ERK (Thr202/Tyr204) antibody (Santa Cruz Biotechnology, Santa Cruz, CA, USA), mouse anti-Rac1 antibody (Upstate Biotechnology, Lake Placid, NY, USA), mouse anti-β-actin antibody (Sigma) and mouse anti-GAPDH antibody (Chemicon, Temecula, CA, USA). Digital images of the immunoblots were taken and analyzed with Quantity One (Bio-Rad, Hercules, CA, USA).

### 3.7. Pull-Down Assays

Rac1 activity was measured as previously depicted [54]. GST-PAK-CRIB (a glutathione S-transferase (GST) fusion protein that contains the Cdc42/Rac-interactive binding region (CRIB) of the p21-activated serine/threonine kinase (PAK), a gift from Dr. James E Casanora, University of Virginia, VA, USA) was used for capturing active Rac1 in cell lysates. Briefly, the GST fusion proteins were purified from *BL21* bacteria and isolated by incubation with MagneGST glutathione particles (Promega, Madison, WI, USA) for 30 min at 4 °C. After treatment of cells with appropriate inhibitors or stimuli, cells were lysed, and the cell lysates were incubated with the particles carrying GST-fusion protein for 1 h on a rotating wheel at 4 °C. The particles were then washed, solubilized in 2× SDS loading buffer and subjected to immunoblotting analysis with anti-Rac1 antibody (Upstate Biotechnology).

### 3.8. Immunofluorescence Staining

Cells used for immunostaining were fixed in 3.7% paraformaldehyde in PBS for 30 min, permeabilized in 0.1% Triton X-100 and blocked in PBS containing 1% BSA for 1 h at room temperature. The cells were then incubated with anti-Rac1 for 2 h. After washes with PBS, the cells were incubated for another 1 h with rhodamine-conjugated anti-rabbit antibody. Next, F-actin was stained with FITC-labeled phalloidin (5 µg/mL) (Sigma) for 40 min. After washing with PBS, the membranes were mounted on glass slides with DAPI Fluoromount G (Southern Biotech, Birmingham, AL, USA). Graphs were obtained using an Olympus BX51 microscope (Olympus, Melville, NY, USA).

### 3.9. Reverse Transcription PCR (RT-PCR)

Total RNA was extracted from cells with TRIzol reagent (Invitrogen). cDNA was synthesized with the SuperScript First-Strand Synthesis kit (Invitrogen) from 1.0-g RNA samples. Quantitative reverse transcriptase PCR (RT-PCR) was carried out using SYBR Green PCR master mix reagents on an ABI 7500 Fast Real-Time PCR System (Applied Biosystems, Foster City, CA, USA). Thermal cycling was conducted at 95 °C for 30 s, followed by 40 cycles of amplification at 95 °C for 5 s, 55 °C for 30 s and 72 °C for 15 s. The relative quantification of gene expression for each sample was analyzed by the delta C_t_ method. The following primers were used to amplify VEGF: 5'-ACCAAGGCCAGCACATAGG-3' (sense) and 5'-ACGCTCCAGGACTTATACCG-3' (antisense); 18S rRNA: 5'-ACCTGGTTGATCCTGCCAGT-3' (sense) and 5'-CTGACCGGGTTGGTTTTGAT-3' (antisense).

### 3.10. Statistical Analysis

Statistical evaluations were performed by the SPSS software. The Student’s *t*-test was used to determine the significance of differences between two groups. One-way ANOVA followed by Student-Newman-Keuls (SNK) tests were employed for multiple comparisons of means. The level of significance was set at *p* < 0.05. 

## 4. Conclusions

In summary, this study highlights the role of ERK/Rac1 in hypoxic-induced HUVEC migration, and has shown that melatonin suppresses cell migration by inactivation of ERK/Rac1 and subsequent HIF-1α expression. These findings are of potential clinical importance for understanding the mechanism of melatonin in suppressing HUVEC motility in hypoxia and may provide new mechanism for the use of melatonin.
